# Task Force for a rapid response to an outbreak of severe acute hepatitis of unknown aetiology in children in Portugal in 2022

**DOI:** 10.2807/1560-7917.ES.2023.28.38.2300171

**Published:** 2023-09-21

**Authors:** Berta Grau-Pujol, João Vieira Martins, Isabel Goncalves, Fernanda Rodrigues, Rita de Sousa, Dina Oliveira, Joana Bettencourt, Diana Mendes, Inês Mateus de Cunha, Sara Pocinho, Ana Firme, Benvinda Estela dos Santos, André Peralta Santos, Maria João Albuquerque, Pedro Pinto-Leite, Rui Tato Marinho, Paula Vasconcelos

**Affiliations:** 1 Center for Public Health Emergencies, Directorate-General of Health, Lisbon, Portugal; 2 Directorate of Information and Analysis, Directorate-General of Health, Lisbon, Portugal; 3 ECDC Fellowship Programme, Field Epidemiology path (EPIET), European Centre for Disease Prevention and Control (ECDC), Stockholm, Sweden; 4 Hospital Pediátrico, Centro Hospitalar e Universitário de Coimbra, Coimbra, Portugal; 5 Faculdade de Medicina da Universidade de Coimbra, Coimbra, Portugal; 6 Infectious Diseases Department, National Institute of Health Doctor Ricardo Jorge, Lisboa, Portugal; 7 Division of Sexual, Reproductive, Child and Youth Health, Directorate-General of Health, Lisbon, Portugal; 8 National Program for Viral Hepatitis, Directorate-General of Health, Lisbon, Portugal; 9 Division of Communication and Public Relationships, Directorate-General of Health, Lisbon, Portugal; 10 Centro Hospitalar Lisboa Ocidental, Lisboa, Portugal; 11 Division of Disease Prevention and Health Promotion, Directorate-General of Health, Lisbon, Portugal; 12 Comprehensive Health Research Centre (CHRC), Escola Nacional de Saúde Pública, Universidade NOVA de Lisboa, Lisboa, Portugal; 13 Centro Hospitalar Universitário Lisboa Norte, Lisboa, Portugal; 14 Faculdade de Medicina, Universidade de Lisboa, Portugal

**Keywords:** acute hepatitis unknown origin, paediatrics, Europe, outbreak response, hospitalisation, cross-border threat, liver transplant, adenovirus

## Abstract

On 5 April 2022, the United Kingdom reported an increase of cases of severe acute hepatitis of unknown aetiology in children, several needing hospitalisation and some required liver transplant or died. Thereafter, 35 countries reported probable cases, almost half of them in Europe. Facing the alert, on 28 April, Portugal created a multidisciplinary Task Force (TF) for rapid detection of probable cases and response. The experts of the TF came from various disciplines: clinicians, laboratory experts, epidemiologists, public health experts and national and international communication. Moreover, Portugal adopted the European Centre for Disease Prevention and Control (ECDC) and the World Health Organization (WHO) case definition and recommendations. By 31 December 2022, 28 probable cases of severe acute hepatitis of unknown aetiology were reported: 16 male and 17 aged under 2 years. Of these cases, 23 were hospitalised but none required liver transplant or died. Adenovirus was detected from nine of 26 tested cases. No association was observed between adenovirus infection and hospital admission after adjusting for age, sex and region in a binomial regression model. The TF in Portugal may have contributed to increase awareness among clinicians, enabling early detection and prompt management of the outbreak.

## Background

On 5 April 2022, the United Kingdom (UK) notified the World Health Organization (WHO) of an increase in severe acute hepatitis cases of unknown aetiology via the International Health Regulations notification system. These 10 cases were previously healthy children. After testing negative for hepatitis virus types A-E, as well as other known factors causing acute hepatitis, the aetiology remained unidentified [1-4].

Acute hepatitis is a rapid inflammation of the liver and can be caused by various factors, including viruses, bacteria, toxins and medicines. Symptoms of acute hepatitis include jaundice, anorexia, fatigue, nausea and vomiting. In rare cases, hepatitis can be a life-threatening disease, leading to liver failure and hepatic encephalopathy and requiring liver transplant to prevent death [5,6].

Considering the situation threat, the European Centre for Disease Prevention and Control (ECDC) and the WHO established a common case definition and reporting protocol for all countries [7]. A probable case was defined as a person presenting with an acute hepatitis (non-hepatitis viruses A-E) with a level of serum alanine or aspartate transaminase > 500 international units (IU)/L, who was 16 years or younger, since 1 October 2021 [8]. Between 5 April and 8 July 2022, 35 countries reported 1,010 probable cases of hepatitis of unknown aetiology in children, 5% required liver transplant and 2% died [8,9].

We describe the outbreak in Portugal and the management and response to it – the constitution and function of a Task Force (TF). The TF in Portugal contributed to communication, case management, data analysis and supported decision-makers of the control measures. Sharing this experience can improve the management and response of future outbreaks.

## Set up and management of the Task Force

**Figure 1:**
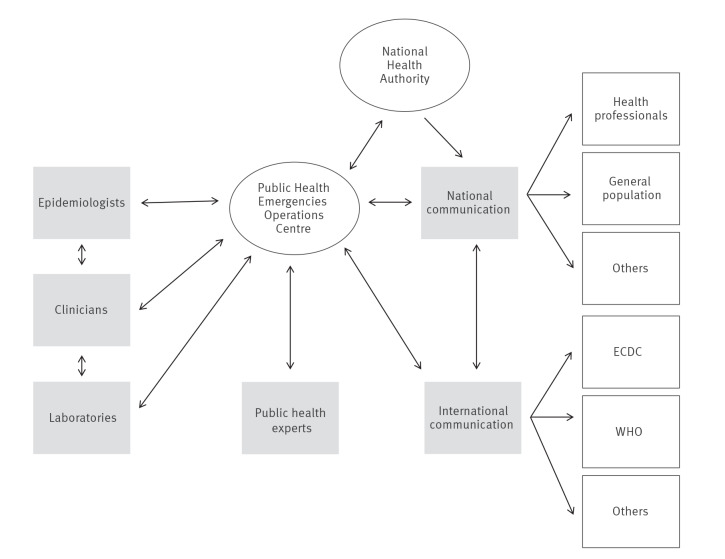
Description of the Task Force for hepatitis of unknown aetiology in children, Portugal, 2022

ECDC: European Centre for Disease Prevention and Control; WHO: World Health Organization.

Upon the detection of the outbreak of hepatitis of unknown aetiology in the UK, the Directorate-General of Health (DGS) of Portugal, through the National Health Authority, established a management and response team on 22 April – the national TF, applying internal procedures to respond to international alerts with potential national impact [10] The TF for hepatitis of unknown aetiology in Portugal consisted of a multidisciplinary team of experts, involving clinicians (paediatricians representing the Portuguese Society of Paediatrics, hepatologists and transplant experts), laboratory experts, epidemiologists and public health and communication experts (Figure 1). The aim of the TF was to increase the capability to early detect and respond to this disease threat. On 26 April, the Portuguese Society of Paediatrics disseminated among paediatricians information on detection and management of hepatitis cases in children.

The TF assessed healthcare, laboratory and paediatric liver transplant capacities and capabilities to ensure early detection of severity and increasing recovery of sick children considering national and international context. The TF gathered international and national evidence and practices to develop new guidelines for early detection, laboratory diagnosis, case notification and case management and regularly updated the guidelines based on the evolution of the outbreak [11]. To collect information about the cases and understand the epidemiological link, an online form was created and subsequently integrated into the supporting application of the Portuguese National Epidemiological Surveillance System (SINAVE). Additionally, the TF maintained a regular appraisal of the epidemiological situation using national and international routine network of alerts (Figure 1).

The TF held regular meetings to follow the evolution of the outbreak, adjusting the frequency as needed (from weekly to monthly). This allowed the TF to understand any potential seasonality of the alert while ensuring timely detection of severe cases.

## Findings of the task force

In Portugal, 32 suspected cases of severe hepatitis of unknown aetiology in children aged < 16 years were notified between 28 April and 30 November 2022. Of those, 28 were identified as probable cases, an incidence of 2 cases per 100,000 children (based on the population of children in Portugal in 2021).

**Figure 2:**
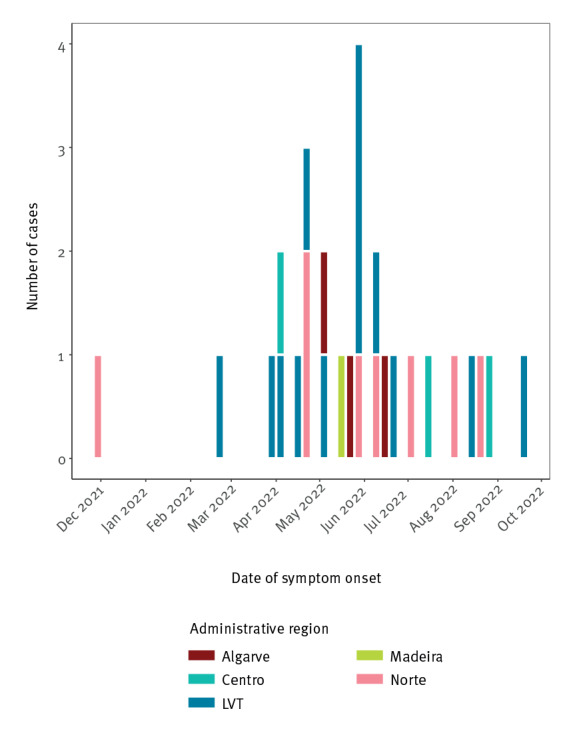
Date of symptom onset of probable cases of hepatitis of unknown aetiology, per region, Portugal, 2022

The first suspected case was notified on 28 April in 2022. A suspected case was a child up to 16 years of age with clinical manifestations suggestive of acute hepatitis (jaundice, anorexia, nausea, intermittent vomiting, choluria and acholia) or with non-specific clinical manifestations (abdominal pain, nausea and vomiting, diarrhoea, with more than 1 week of evolution and significant prostration or with respiratory symptoms and fever). The first probable cases (n = 8) were notified on 12 May 2022: symptoms of one case started on 30 November 2021 and seven presented symptoms between February and April 2022 (Figure 2). The latest probable case was notified on 25 October 2022, with onset of symptoms on 18 September 2022.

LVT: Lisboa and Vale do Tejo.

Sixteen of the 28 cases were male and 17 were younger than 2 years. Most cases were reported from the two most populated regions: almost half from the region of Lisboa and Vale do Tejo (n = 13), followed by the Norte region (n = 8). No cases were epidemiologically linked. Additional information can be seen in Supplementary Table S1.

## Disease severity

None of the 28 probable cases notified between 28 April and 30 November 2022 required liver transplant or died. Nevertheless, 23 probable cases were hospitalised. The median length of hospital stay was 5 days (range 1-35 days). More details can be seen in the Supplementary Figure S1.

**Figure 3:**
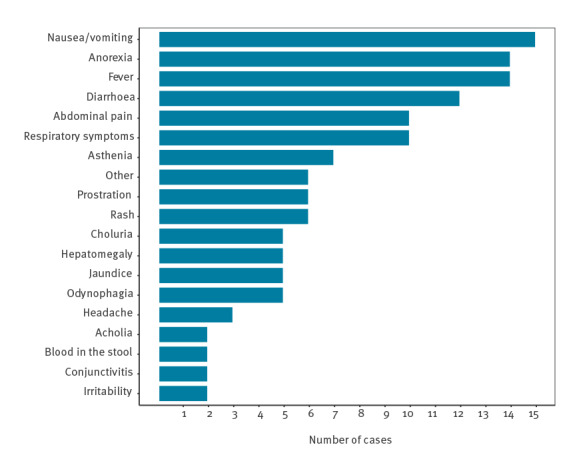
Symptoms of probable cases of hepatitis of unknown aetiology in children Portugal, 2022 (n = 28)

The most common symptoms were fever (n = 14) and gastrointestinal symptoms, including nausea and vomiting (n = 15), anorexia (n = 14), diarrhoea (n = 12) and respiratory symptoms (n = 10) (Figure 3). Gastrointestinal and respiratory symptoms were observed in all ages.

The median of the highest value measured for alanine transaminase was 1,050 IU/L (min: 415 IU/L, max: 4,126 IU/L) and for aspartate transaminase was 1,163 IU/L (min: 166 IU/L, max: 5,367 IU/L). More information can be seen in the Supplementary Figure S2.

**Figure 4:**
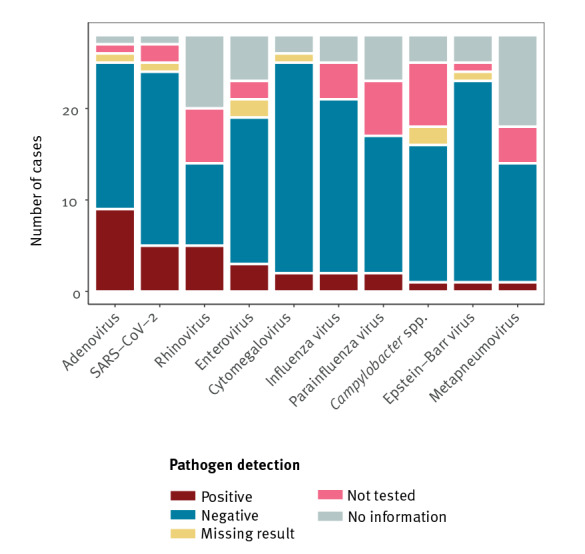
Microbiological analyses of samples from probable cases of hepatitis of unknown aetiology in children, Portugal, 2022 (n=28)

Adenovirus was detected by real-time PCR from stool samples of nine of 26 probable cases tested. Four adenovirus isolates were genotyped at the national reference laboratory (National Institute of Health, INSA): human adenovirus (HAdV) species C (HAdV-C) was detected in two and HAdV-F41 in two isolates. Testing of nasopharyngeal swabs by real-time PCR yielded SARS-CoV-2 from five of 25, a rhinovirus from five of 14 and an enterovirus from three of 21 probable cases tested (Figure 4). No association was observed between any of the pathogens detected and hospitalisation, after adjusting for age, sex and region in a binomial regression.

SARS-CoV-2: severe acute respiratory syndrome coronavirus 2.

## Response measures

Portugal adopted the ECDC/WHO case definition and recommendations. According to those, urgent hospital admission was required for children up to 16 years of age with clinical manifestations suggestive of acute hepatitis or nonspecific clinical manifestations such as abdominal pain, nausea and vomiting, diarrhoea, jaundice, with more than 1 week of evolution and significant prostration. Moreover, laboratory analyses were requested according to ECDC/WHO guidelines [7]. Probable cases were required to be promptly notified to the DGS. As part of the notification, clinical doctors encountering a patient who met the probable case definition had to complete an online SINAVE form. Epidemiological information on each case, e.g. location of the case, educational centre or contact with other sick children were included in the form to facilitate the TF discern potential epidemiological links among the cases. Moreover, the TF reinforced finding of retrospective cases among paediatricians through the Portuguese Society of Paediatrics. All cases were discussed and validated within the TF to evaluate if the criteria for probable case were met and to ensure that microbiological findings of all cases were confirmed at the National Reference Laboratory. These discussions between clinicians, epidemiologists and laboratory experts ensured complementary laboratory investigation, including adenovirus genotyping when isolates were available.

Patients with acute hepatitis that did not meet probable case criteria were followed-up.

In addition, the TF recommended prevention and control measures for suspected or probable cases in healthcare facilities, complying with established rules for infection control by the National Programme for the Prevention and Control of Infections and Antimicrobial Resistance (PPCIRA).

The TF communicated to the media and to healthcare professionals on protective measures, such as hand hygiene (with supervision of younger children), ventilation of interior spaces, frequent cleaning and/or disinfection of surfaces in facilities with cases of acute gastroenteritis or respiratory infection [11]. The aim was to share the latest scientific evidence available in a transparent and approachable language, to avoid panic.

## Discussion

We described the constitution, management, and response of the TF in Portugal, following the international alert of severe acute hepatitis of unknown aetiology in children in April 2022. The set-up and function of the national TF was timely and satisfactory: probable cases were discussed at the first TF meeting, guiding and supporting management of cases with early symptoms.

The composition of the TF facilitated multidisciplinary interaction and collaboration. Moreover, it provided tailored evidence-based information to all audiences, guiding clinical doctors and mitigating fear among the general population. A multidisciplinary approach was also pursued in other countries and the effect is supported by other publications [12-14]. In the UK, the TF also included clinical, virology and genomics researchers [3].

The age and sex distribution of the cases was similar to other countries. Nevertheless, the cases in Portugal were detected slightly later. The WHO Regional Office for Europe (WHO/Europe) described probable cases from January 2022, and case numbers started to peak in March [15], whereas only two probable cases were described before April in Portugal and the case numbers peaked in June. This delay could be related to seasonal factors. Additionally, although the TF successfully reinforced retrospective case finding (symptoms of 8 of 28 notified probable cases started 1-5 months before the set-up of the TF), some probable cases could have been missed.

None of the cases in Portugal required liver transplant or died, while these outcomes were observed in other countries in Europe and elsewhere [9,15]. As in Portugal, most of the outbreak cases were hospitalised [16,17]. The median duration of hospital stay (5 days) was slightly longer than in other countries in the case of no liver failure [18], which could possibly be explained by different criteria for hospital admission and discharge [19]. The symptoms of the cases in Portugal were also slightly different: jaundice, pale stools and hepatomegaly were not as frequent as in the UK [3]. Around a third of the probable cases were infected with adenovirus, fewer than seen in other EU countries and the UK, and a fifth by SARS-CoV-2, higher than observed in other EU countries [3,15]. Nevertheless, infection with adenovirus or SARS-CoV-2 was not associated with hospitalisation in Portugal. While many probable cases were hospitalised (23 of 28), not all were tested for all pathogens: medical doctors might consider testing unnecessary if the patient is recovering. Moreover, sampling of discharged probable cases was mostly logistically and economically unfeasible. However, even though these viruses may contribute to severe acute hepatitis, results from case-control and cross-sectional studies are contradictory [3,12]. In this case, this multi-country outbreak of hepatitis of unknown aetiology might be associated with adenovirus infection and involve a genetic predisposition [20]. In light of the findings by Perrocheau et al. (2023), more than one event occurring concurrently could lead to atypical epidemiology in an outbreak [13]. Thus, further clinical, microbiological, histological and molecular markers, especially from potential new probable cases, are still necessary to improve the characterisation of the outbreak and to compare events among countries.

A similar structure as in the TF described here was formed to respond to mpox in Portugal in May 2022. This TF was still active and able to timely detect mpox cases in the spring of 2023. We consider that sustained investments in the core public health system are essential for early detection of cases and to minimise severe outcomes, especially encompassing the last challenges confronting public health - outbreaks of new diseases or of unknown aetiology or diseases with unusual characteristics. We consider that the presented approach could contribute to guide the management and response to any future cross-border outbreak at early stage, ensuring awareness and rapid response to any public health threat.

## Supplementary Data

90000 Supplementary Material 1
