# Supplementary material for: Task Force for a rapid response to an outbreak of severe acute hepatitis of unknown aetiology in children in Portugal in 2022
Source: Euro Surveill. 2023 Sep 21;28(38):2300171. doi: 10.2807/1560-7917.ES.2023.28.38.2300171 (PMC10515495; doi:10.2807/1560-7917.ES.2023.28.38.2300171)

Supplement

This supplementary material is hosted by *Eurosurveillance* as supporting information alongside the article ***Task Force for the rapid response to the outbreak of severe acute hepatitis of unknown aetiology in children in Portugal in 2022***, on behalf of the authors, who remain responsible for the accuracy and appropriateness of the content. The same standards for ethics, copyright, attributions and permissions as for the article apply. Supplements are not edited by *Eurosurveillance* and the journal is not responsible for the maintenance of any links or email addresses provided therein.

Supplementary Table S1 Descriptive characteristics of probable cases with severe hepatitis of unknown aetiology during the outbreak in 2022 in Portugal.

		Number of cases	
		n	%
Sex	Female	12	42.9
	Male	16	57.1
Age (years)	< 1	8	28.6
	1-2	9	32.1
	3-4	3	10.7
	5-10	5	17.9
	11-16	3	10.7
Region of diagnosis	ARS Açores	0	0.0
	ARS Alentejo	0	0.0
	ARS Algarve	3	10.7
	ARS Centro	3	10.7
	ARS Lisboa e Vale do Tejo	13	46.4
	ARS Madeira	1	3.6
	ARS Norte	8	28.6
Clinical condition	Outpatient visit	28	100.0
	Hospital admission	23	82.1
	Transplant	0	0.0
	ICU		0.0
	Death	0	0.0

ARS - Administração Regional de Saúde - Regional Health Administration
UCI – Intensive Care Unit

Supplementary Figure S1 Days of hospital admission of the provable cases of hepatitis with unknown aetiology per age group during the outbreak in 2022 in Portugal.

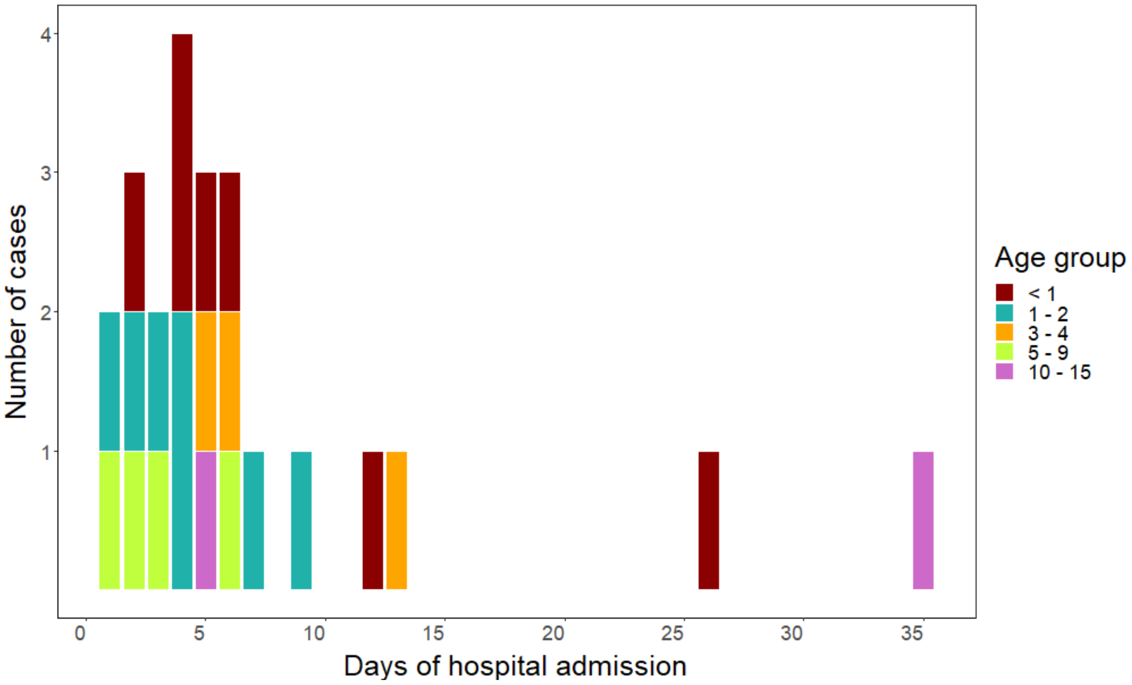

Supplementary Figure S2 Highest value of the aspartate and alanine transaminases (UI/L) that presented the probable cases of hepatitis with unknown aetiology during the outbreak in 2022 in Portugal. Dotted line displays the 500UI/L threshold.

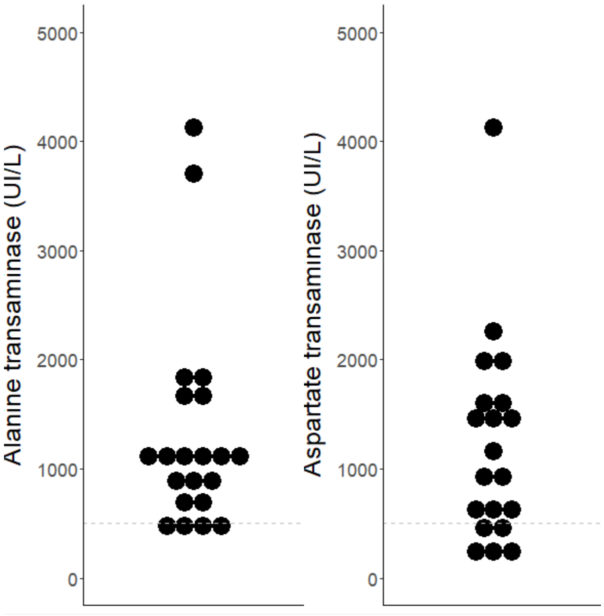

Supplement: Supplementary Material 1 [file 23-00171_GRAU-PUJOL_Supplement.pdf]
